# Is *Shewanella oncorhynchi* a fish health threat? Possible genetic background of pathogenicity and common carp challenge

**DOI:** 10.2478/jvetres-2025-0042

**Published:** 2025-08-20

**Authors:** Ewa Paździor, Agnieszka Pękala-Safińska, Arkadiusz Bomba, Dariusz Wasyl

**Affiliations:** 1 Department of Parasitology and Invasive Diseases, Bee Diseases and Aquatic Animal Diseases, Department of Research Support, Department of Bacteriology and Bacterial Animal Diseases, National Veterinary Research Institute, 24-100 Puławy, Poland; 2 Department of Preclinical Sciences and Infectious Diseases, Faculty of Veterinary Medicine and Animal Science, Poznań University of Life Sciences, 60-637 Poznań, Poland; 3 Department of Research Support, Department of Bacteriology and Bacterial Animal Diseases, National Veterinary Research Institute, 24-100 Puławy, Poland; 4 Department of Bacteriology and Bacterial Animal Diseases, National Veterinary Research Institute, 24-100 Puławy, Poland

**Keywords:** carp epithelial cells, challenge tests, cytotoxicity, virulence genes, whole genome sequencing

## Abstract

**Introduction:**

The *Shewanella* genus is widely distributed in aquatic environments and occurs in different niches with a wide range of temperatures and salinities. It includes a novel species, *S. oncorhynchi*, described as recently as 2022, causing lens atrophy, inappetence and growth retardation in rainbow trout (*Oncorhynchus mykiss*, Walbaum 1792). *Shewanella oncorhynchi* also occurs in common carp (*Cyprinus carpio* L.), but its potential pathogenicity in this species is unclear.

**Material and Methods:**

In this study, the pathogenicity of three well-characterised *S. oncorhynchi* strains was assessed *in vivo* in experimental infection of common carp and *in vitro* by estimation of their cytotoxicity to an epithelioma papulosum cyprini cell line. The strains’ characterisation involved whole-genome sequencing to identify possible virulence genes.

**Results:**

Our study proved the pathogenicity of *S. oncorhynchi* to common carp and the bacterium’s cytotoxicity to epithelioma papulosum cyprini cells. Swollen abdomens, lens opacity, areas of discoloration and skin lesions were recorded in infected common carp. However, the ability to cause disease symptoms and mortality depended on the strain.

**Conclusion:**

The study showed the potential roles of the quorum-sensing system, type IV pili, fimbriae, stress survival, iron metabolism and secretion system genes in the virulence of *S. oncorhynchi*. Of these, the *vscC2, vscN2, vscR2, vscS2* and *vscU2* type III secretion system genes potentially and probably enhance the bacterium’s ability to cause lesions in common carp.

## Introduction

*Shewanella* spp. belong to the *Gammaproteobacteria* class and *Alteromonadales* order and consist of a group of Gram-negative bacteria that widely inhabit freshwater and marine environments from the surface to the deepest parts of sediment (12, 19). The wide distribution of *Shewanella* species is associated with their adaptability to growing in different niches, especially in a wide range of temperatures and salinities (19). Some species have been recognised as causative spoilage agents of certain proteinaceous foods, particularly fish even when stored at low temperature (35). *Shewanella* spp. can also cause human diseases: mostly skin and soft tissue infections but also ophthalmological or intracranial infections, pneumonia and endocarditis (23, 24, 36).

The first isolation of a member of the *Shewanella* genus from cultured freshwater diseased fish was reported in 2004 (17). Since then, numerous cases of infection with *Shewanella* species have been described worldwide in freshwater fish belonging to the *Salmonidae, Cyprinidae, Acipenseridae, Anguillidae, Cichlidae* and *Centrarchidae* families (1, 7, 8, 9, 11, 13, 14, 27, 28, 31, 32).

*Shewanella putrefaciens, S. baltica, S. oneidensis* and *S. xiamenensis* have been isolated from common carp (*Cyprinus carpio* L.), the dominant freshwater fish reared in central and eastern Europe (14, 17, 28). These isolations involved diagnosis based on bacterial culture, phenotypic characterisation and/or 16S rRNA sequencing. Whole-genome sequencing showed that the recently described *S. oncorhynchi* was also isolated from common carp (2, 26). In rainbow trout (*Oncorhynchus mykiss*, Walbaum 1792), *S. oncorhynchi* caused lens atrophy, inappetence, lethargy, reduction of feed intake and growth retardation (2, 32). This bacterium is related to *S. hafniensis* and *S. baltica* (2, 26), and comprehensive genome analyses revealed that like these species, it also carried the genes responsible for protein secretion systems, for quorum sensing systems and for enterotoxin production, which may contribute to its virulence (2, 32).

Our previous analysis showed a considerable share of *S. oncorhynchi* in fish infections (26). Therefore, the current study aimed to evaluate the *in vivo* and *in vitro* virulence of pre-selected strains carrying the genes possibly involved in the pathogenicity of *S. oncorhynchi*.

## Material and Methods

### Bacterial strains

Of the 35 strains of *S. oncorhynchi* characterised previously (26), 3 strains were selected for the current study to determine the pathogenicity of this bacteria for common carp. Initially, 22 strains from common carp were taken into consideration, and analyses based on source and their properties were performed, particularly targeted on establishing phylogenetic relationships and the presence of potential virulence genes. Finally, strains representing three gene profiles were arbitrarily selected: K999, K313 and K61. Their genes which possibly impact pathogenicity are shown in Table 1.

**Table 1.** Potential virulence genes of the *Shewanella oncorhynchi* strains

Function	Gene	K61	K313	K999
Flagella protein	*flgI flmH fleN*/*flhG motA fliG fliM fliP flhA cheW cheY*	+	+	+
	*cheV*		+	
	*lfgG*		+	+
Type IV pilus protein	*tapC tapT tufA mshG*	+	+	+
	*tapB*			+
Heat-shock protein	*htpB*	+	+	+
T2 secretion system	*exeF*			+
	*epsE epsG*	+	+	+
T3 secretion system	*vscC2 vscN2 vscR2 vscS2 vscU2*		+	+
Ferric-siderophore adenosine	*bauB*			+
5′-triphosphate binding cassette transporter	*bauD*	+	+	+
Immune modulation	*galE pseC rpoS lpxC kdsA*	+	+	+
	*rfbC rffG*			+
	*ugd pseI*		+	
	*wbtL ACICU cap8E*	+		
Regulation	*rpe tufA csrA fur*	+	+	+
Stress survival	*katA clpP sodB*	+	+	+
Biofilm	*adeG algU luxS*	+	+	+
Putative carbonic anhydrase	*mig-5*			+

### Challenge tests

Before the experiment, 165 common carp of 90±10 g weight were acclimatised to laboratory conditions in a tank of 1000 L volume. Five randomly selected fish were examined negative for possible parasitological and microbiological agents. During all procedures, the fish were kept at a temperature of 14±1°C, in water containing 6±0.5 mg/L of dissolved oxygen and flowing at 15 L/h, and in a 12/12-h light/dark cycle. The fish were fed with a nutritionally balanced feed suitable for common carp (Aller Aqua, Golub-Dobrzyn, Poland). No health disorders or mortality were observed during the animal acclimatisation period.

After 14 days of acclimatisation, the fish were divided into three experimental groups (A, B and C) and a control group (K). Each group of 40 fish was placed in a tank of 200 L volume. Individuals from each experimental group were injected intraperitoneally with 0.5 mL of one selected strain (group A’s challenge was with K999, group B’s with K313 and group C’s with K61) at a concentration of 10^8^ colony-forming units/mL per fish, while the control group fish were injected with 0.5 mL of phosphate-buffered saline (PBS). The procedures were performed after the fish were anaesthetised by immersion using MS-222 water solution (150 mg/L; Sigma-Aldrich, St. Louis, MO, USA).

The fish were observed daily for any clinical signs over a 21-day period. Fish from each group were sacrificed to collect internal organs. Kidneys and lesion tissues either from those individuals or from daily moribund fish were collected. Bacteriological methods, including biochemical properties and enzyme activity, were performed as described previously (27).

### Genome sequencing

Three *Shewanella* strains isolated from kidney samples of each experimental group (A, B and C) were whole-genome sequenced. Extraction of genomic DNA was performed using the Genomic Mini kit (A&A Biotechnology, Gdańsk, Poland) according to the manufacturer’s instructions. Whole-genome sequencing was performed using the MiSeq platform (Illumina, San Diego, CA, USA). Library preparation, sequencing, quality control and assembly were carried out as described previously (26). To compare original challenge strains with their reisolated strains obtained from experimental fish samples, fastq files were trimmed using fastp 0.23.2 (6). Next variant calling analysis was performed by freebayes 1.3.2 (10), and detected single-nucleotide polymorphisms (SNPs) were filtered with minimum depth ≥10× and allele frequency >90%.

The presence of potential virulence genes in the bacterial genome assembly was determined using Abricate 1.0.1 (33) against the Virulence Factor Database (5). The analyses only included genes of which the similarity to genes contained in the tested bacteria was ≥60% and sequence coverage ≥60%.

### *In vitro* virulence assay

The cytotoxicity of *Shewanella oncorhynchi* to the epithelioma papulosum cyprini (EPC) cell line was estimated by measuring lactate dehydrogenase (LDH) release activity in the damaged cells using the CyQUANT LDH Cytotoxicity Assay Kit (Invitrogen, Carlsbad, CA, USA). Before the tests, the optimal number of cells was determined according to the manufacturer’s instructions. The preliminary density of the EPC cell line was about 3 × 10^5^/mL. The absorbance values for the spontaneous release of LDH from cells (spontaneous LDH) and the activity of the enzyme released from chemically lysed cells (maximum LDH) were analysed across different EPC cell densities (Supplementary Fig. S1). The number of EPC cells for cytotoxicity testing was established at 1.5 × 10^5^/mL. The optimal incubation time for EPC cells with bacteria was 48 h. Longer incubation caused an increase in the level of LDH in no infected cells, which indicated spontaneous cell death (Supplementary Fig. S2).

Strains were cultured on tryptic soy broth (BioMérieux, Marcy-l’Étoile, France) at 24±2°C for 24 h and washed three times in PBS free from calcium and magnesium cations (Biomed, Lublin, Poland) by centrifugation at 2,000 × *g* and 4°C for 15 min. Each bacterial strain was individually added to appropriate-density EPC cells in a 96-well culture plate at the concentration of 10^8^ bacterial cells per well. Spontaneous LDH and maximum LDH activity were included in each experimental plate. Following this, incubation was carried out for 48 h at 24±2°C. The infected cell cultures were observed under an inverted microscope after 2 h, 24 h and 48 h. Lactate dehydrogenase activity was determined using a UV-VIS 5100 spectrophotometer (Metash Instruments, Shanghai, China) by measuring the absorbance of each well at 490 nm and subtracting the 680 nm absorbance value (the background signal from the instrument).

The percentage of cytotoxicity was calculated for 48-h incubation based on sample LDH activity, spontaneous LDH and maximum LDH released from the chemically lysed cells according to the instructions provided by the manufacturer of the CyQUANT Cytotoxicity Assay Kit (Invitrogen). The cytotoxicity of *S. oncorhynchi* to EPC cells was determined in triplicate experiments to obtain the most precise results.

## Results

### Challenge tests

**Fig. 1.**
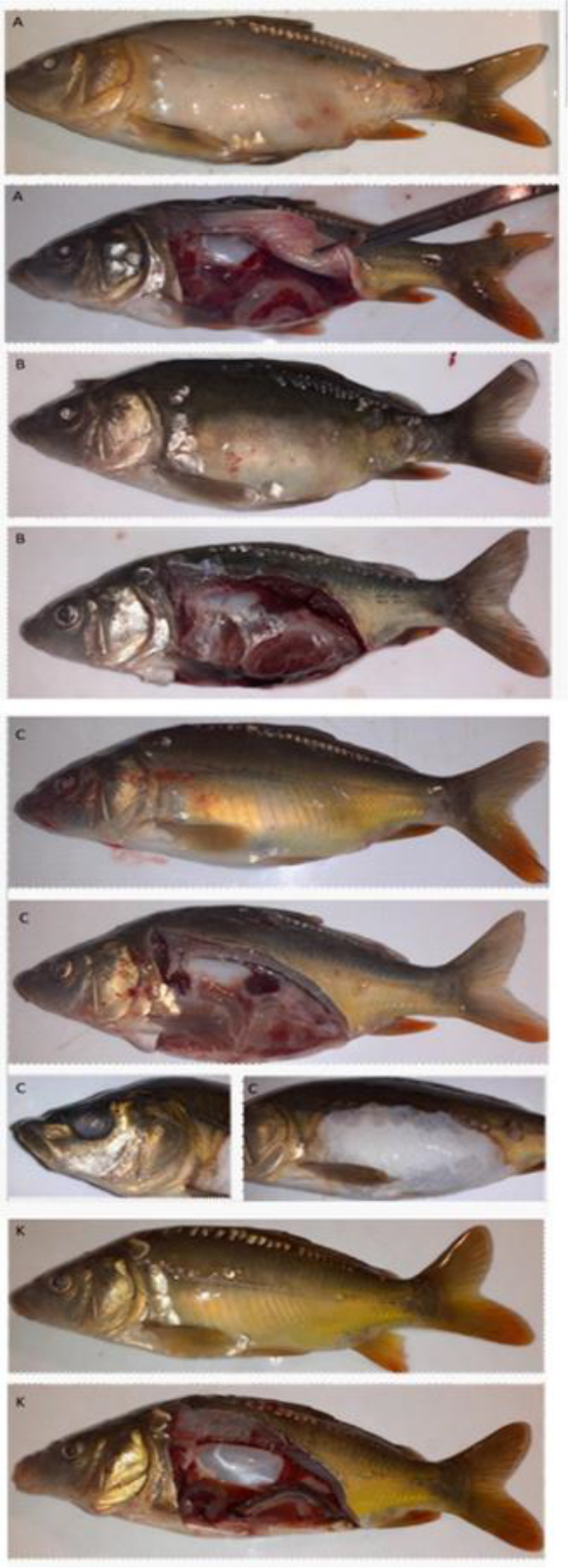
Lesions observed in common carp challenged with *Shewanella oncorhynchi*: group A (bacterium strain K999) – swollen abdomens, discolorations of the skin, lens opacity and swelling of the kidneys; group B (strain K313) – slightly swollen abdomens, discolorations of the skin, lens opacity and swelling of the kidneys; group C (strain K61) – slightly swollen abdomens, lens opacity, swelling of the kidneys, skin lesions and discolorations; group K – no lesions

**Fig. 2.**
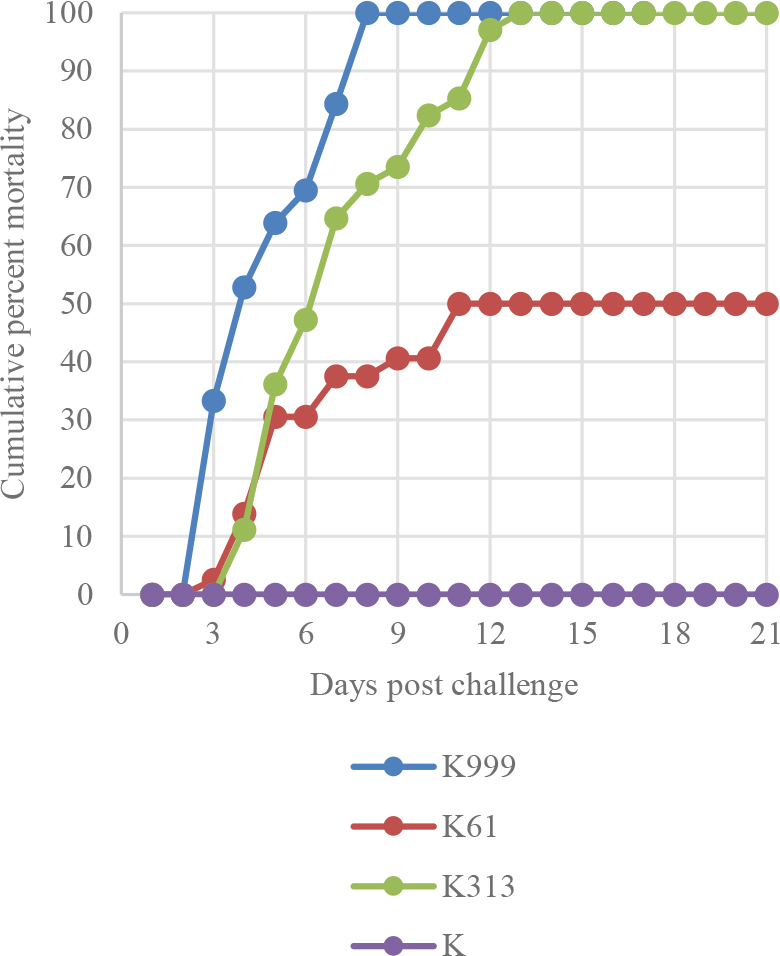
Cumulative mortality in fish infected with K999, K313 and K61 strains and in uninfected fish

During the experiment, no health disorders or mortality were observed in fish from the control group (K). The first clinical signs of health disorders in experimental group fish occurred no earlier than 3 d after injection. In group A infected with the K999 strain, poor food intake, swollen abdomens, discolorations of the skin, and, in some animals, lens opacity were observed (Fig. 1), followed by death. On the eighth day of the experiment, the mortality of group A fish reached 100%. In common carp infected with the K313 strain (group B), the first clinical signs appeared on the fifth day post infection. The only symptoms different to those noted in group A fish were discolorations and slight skin lesions affecting the pelvic and anal fins. Mortality also affected all group B fish, but later than it did the group A fish, on the 13^th^ day after infection (Fig. 2). The clinical picture in common carp infected with the K61 strain (group C) differed: poor food intake was noted on the sixth day after injection, and slightly swollen abdomens on day 10. Also, discolorations of the skin and small ulcers were observed in two fish near the experiment end, and lens opacity was noted in only a single animal on day 16. The overall group C fish mortality rate was 47.5%.

In the post-mortem examination of the fish that had died after having been observed moribund, swelling of the kidneys and the presence of exudative fluid in the body cavity were noted in specimens from all experimental groups. In some cases intestinal hyperaemia was also observed. Samples of kidneys collected from all experimental groups on day 7 after injection were positive for *Shewanella*. In addition, the bacterium was isolated from lesions of the skin and eye. No bacteria were detected in the control group.

Based on biochemical properties, all strains obtained from infected fish were perfectly identified as being in the *S. putrefaciens* group and with distinct biochemical profiles as matched by API 20E (27):0502000 (K999), 0402004 (K313) and 0502004 (K61). Strain K999 decarboxylated ornithine, in contrast to K313 and K61. Positive enzymatic reactions for esterase (C4), esterase lipase (C8), alkaline phosphatase, leucine-arylamidase, trypsin, α-chymotrypsin, alkaline phosphatase, naphthol-AS-BI-phosphohydrolase, and N-acetyl β-glucosaminidase were observed in all strains. No biochemical or enzymatic properties differed between the original strains and the re-isolated ones.

### Genome sequencing

The whole-genome sequences of the three *S. oncorhynchi* strains revealed 51 putative virulence genes, of which 28 were found in strain K999, 25 in K313 and 21 in K61 (Table 1). Different chromosomal genes related to flagella (*cheV, cheW, cheY, flmH, lfgG, fliG, fliM, fliP, fleN/flhG, flgI, flhA* and *motA*), fimbriae (*tapB, tapC, tapT, tufA* and *mshG*), heat shock protein (*htpB*), secretion system proteins (*exeF, epsE, epsG, vscC2, vscN2, vscR2, vscS2* and *vscU2*), the ferric siderophore adenosine 5′-triphospate binding cassette transporter (*bauB* and *bauD*), immune modulation or regulation (*rfbC, rffG, galE, ugd, pseC, pseI, rpoS, lpxC, kdsA, wbtL, ACICU_RS00475, cap8E, rpe, csrA, fur* and *tufA*), stress survival (*katA, clpP* and *sodB*), biofilm formation (*adeG, algU* and *luxS*) and putative carbonic anhydrase (*mig-5*) were detected. Those genes were most similar to the virulence genes deposited in the Virulence Factor Database, with coverage percentages ranging from 61.8 to 100.0%. Comparison of the original challenge strain sequences and the reisolated strains from carp tissue samples showed no SNP differences between multiple K999, K313 and K61 strains. Respective sequences were deposited in the European Nucleotide Archive under the accession Nos ERR10850092, ERR10850099 and ERR10850106.

### *In vitro* virulence assay

**Fig. 3.**
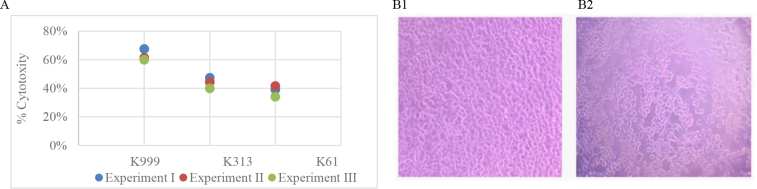
Cytotoxicity of *Shewanella oncorhynchi* to common carp epithelial cells: A – Percent of cytotoxicity of K999, K313 and K61 strains after 48 h incubation; B1 – Photomicrograph (×100) of carp epithelial cells uninfected with any strain of *S. oncorhynchi* (control groups cells); B2 – Photomicrograph (×100) of carp epithelial cells infected with K999 strain. Experiment I – initial 48-h experiment; Experiment II – first independent repeat; Experiment III – second independent repeat

As shown in Fig. 3, all tested bacteria were cytotoxic to EPC cells. Similar results were obtained in two independent repeats. A cytopathic effect was observed compared to the control cells. Changes in the morphology of the infected cells and degeneration due to nucleus and cytoplasmic vacuolation were detected.

## Discussion

In this study, we proved the *in vivo* pathogenicity of *S. oncorhynchi* to common carp. Swollen abdomens, areas of skin discolourations and, lesions, lens opacity, swelling of the kidneys and the presence of exudative fluid in the body were developed. As similar findings to ours, in the only described case of disease caused by *S. oncorhynchi* in rainbow trout, lens abnormalities were reported (32). In both the present research and the research on rainbow trout infection, bacteria were isolated from the eye and kidney. However, unlike our results, those of Saticioğlu *et al*. (32) do not describe other disorders or internal organ lesions, nor any mass mortality. Therefore, it is worth considering whether the clinical symptoms caused by *S. oncorhynchi* depend on the host species. The results of the *in vivo* challenge showed that disorders in common carp were observed regardless of the challenging *S. oncorhynchi* strain; however, the time of appearance of the first symptoms of the overall disease varied. The first signs occurred most rapidly in fish infected with the K999 strain on day 3, and fish infected with the K313 and K61 strains presented signs only on the fifth and sixth days, respectively. Nevertheless, 100% mortality was observed in fish infected with K999 and K313 , up to two weeks post challenge. In contrast, half of the fish survived the exposure to K61. Our observation may indicate that *S. oncorhynchi* is pathogenic for carp, but the pathogenicity and virulence vary from strain to strain.

This variability is in contrast to the outcome of our *in vitro* experiment, which proved that all the three *S. oncorhynchi* used for the fish challenge did develop a cytotoxic effect on EPC cells. This is in line with the findings of Jung-Schroers *et al*. (14) who reported the *in vitro* capacity of the bacterium to damage epithelial cells; however, they also noted no significant difference between tested strains. This might be understood in a clinical context, recognising that epithelial cells serve as the primary barrier against pathogens. It would be predictable that all challenge strains were able to affect the epithelium, but not that further generalised infection had to be similar. Such a concept of bacterial–host interactions is supported by scientific literature (18, 34).

Our results provide insightful data regarding the presence of potential virulence genes. They indicate genetic differences between *S. oncorhynchi* strains and the presence of genes encoding putative virulence factors. All the three currently tested strains contained the quorum-sensing system *luxS* gene related to the production of autoinducer-2 (AI-2), as reported in other studies (32, 37). The quorum-sensing system plays a role in inter-species communication and biofilm formation and occurs in most *Shewanella* species (4, 21). In *S. baltica*, a species closely related to *S. oncorhynchi*, the AI-2 activity intensified with increases in cell density (38). Cerbino *et al*. (4) reported potential virulence genes related to type IV pili, fimbriae, iron metabolism and stress survival which were characteristic of most *Shewanella* species. These genes were detected in all tested strains in the present study. Initiation of the infections caused by many bacterial fish pathogens is related to pili or fimbriae, organelles of adhesion allowing bacteria to colonise skin or mucosal surfaces (22, 29). The role of iron acquisition mechanisms in virulence has not yet been clearly explained in fish pathogens; however, a bacterial pathogen’s ability to infect a host is dependent on its capacity to acquire iron from the environment (20). The stress survival genes in fish pathogens have not been clearly defined, but they are involved in the survival of bacteria under stress conditions (15). The genes related to the quorum-sensing system, type IV pili, fimbriae, stress survival and iron metabolism were characteristic of the *Shewanella* genus; therefore, their roles in the pathogenicity of *S. oncorhynchi* probably are in the strain’s survival in the environment, adhesion to the host and initiation of infection in stress conditions. The mimic occurrence of the above determinants might result in variable experimental disease picture.

Bacterial fish pathogens transfer virulence factors across the membrane either to the extracellular environment or into host cells, using different secretion systems for each destination (22). The genes encoding these factors which specifically related to the type III (T3SS) and II (T2SS) secretion systems were indicated to differ between the tested strains of *S. oncorhynchi* in our study. Potential virulence genes related to the T3SS, namely *vscC2, vscN2, vscR2, vsc*S2 and *vscU2* similar to those of *Vibrio parahaemolyticus*, were detected in two out of the three strains tested in the present study. These strains, K999 and K313, caused higher mortality in infected fish than the strain without this gene, K61. Our study suggested that the type III secretion system is probably the most important factor for infectivity in *S. oncorhynchi*. In agreement with our study, in *S. oncorhynchi* strain S-1 causing lens atrophy in rainbow trout, T3SS genes were also detected (32). Interestingly however, among 144 genomes of *Shewanella* spp. analysed by Cerbino *et al*. (4), the *vscC2, vscN2, vscR2, vsc*S2 and *vscU2* potential virulence genes were detected only in six, one of which was the genome of strain WE21 classified to *S. oncorhynchi* based on whole-genome sequencing. The type III secretion system constitutes a way for bacteria to manipulate the physiology of the host cells and plays an important role in the virulence of many bacterial pathogens of fish and other aquatic animals (30). Nevertheless, within the *Shewanella* genus, T3SS was noted to be considerably more frequent in the genome of one particular species, which indicates the need for further research on *S. oncorhynchi* into the regulatory mechanisms of the expression of these genes and into the secretion of bacterial proteins into fish cells (25).

The other secretion-related component of the genome coding for virulence is the T2SS. It delivers toxins or a wide range of enzymes such as proteases and is important for the survival for opportunistic pathogens finding their primary niche outside the host. Genes of this system were also detected in most *Shewanella* species (3, 4, 16). All three strains tested in our study contained the *epsE* and *epsG* potential virulence genes, while only strain K999, the most virulent, harboured *exeF*. Therefore, the secretion of different extracellular factors may determine the pathogenicity of particular strains; however, it requires further analysis.

Also pertaining to virulence, only in strain K999 did our study confirm the presence of the *mig*-5 gene, putatively one for virulence, encoding carbonic anhydrase. This was a similar finding to one in a previous report (32), which correlated the presence of the *mig*-5 gene with pathological findings observed in infected rainbow trout.

Finally, we indicated that *S. oncorhynchi* showed high enzymatic activity, which was also described in the previous research cited above (32). The similar biochemical profiles of the three tested strains were consistent with the properties of *S. oncorhynchi* strain S-1 isolated from rainbow trout which were reported by Saticioğlu *et al*. (32). In contrast to our results, strain S1 does not hydrolyse β-glucosidase and esculin. Interestingly, ornithine decarboxylation was observed only in strain K999 in contrast to K313, K61 and the reference S1 strain.

## Conclusion

The pathogenicity of various strains of *S. oncorhynchi* to common carp was confirmed by *in vivo* challenge and demonstrable cytotoxicity to epithelioma papulosum cyprini cells. To the best of our knowledge, this study is the first report of the pathogenicity of the recently described *S. oncorhynchi* to this fish species. The three tested strains of *S. oncorhynchi* were demonstrated to have diverse profiles of the genes that cause fish disorders; ability to cause disease may be thought to depend on the specific strain. Virulome analysis showed that *S. oncorhynchi* encoded different genes which may play a role in virulence. Further studies need to be undertaken to better understand the pathogenesis of the disease caused by this bacterium in common carp.

## Supplementary Material

Supplementary Material Details
