# Supplementary material for: Is Shewanella oncorhynchi a fish health threat? Possible genetic background of pathogenicity and common carp challenge
Source: J Vet Res. 2025 Aug 20;69(3):331–7. doi: 10.2478/jvetres-2025-0042 (PMC12503224; doi:10.2478/jvetres-2025-0042)
Supplement: Supplementary file 1 — Supplementary Material Details [file jvetres-2025-0042_sm.pdf]

Supplementary Material

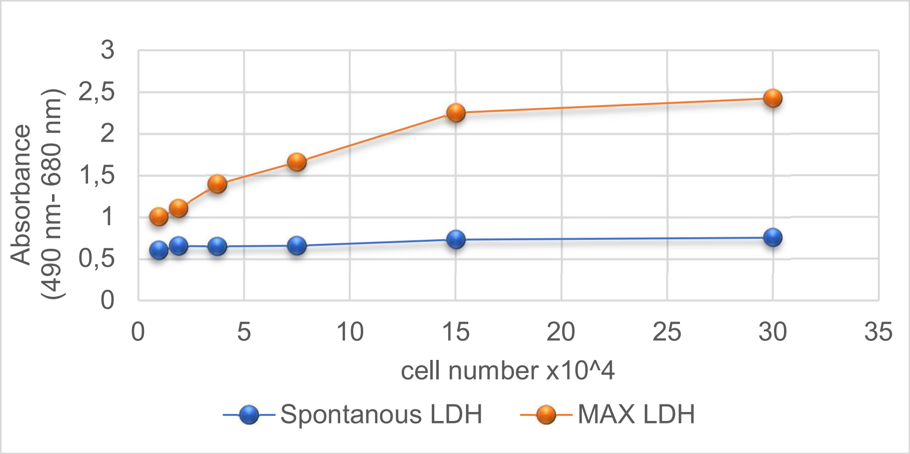

Supplementary Fig. S1. The absorbance values for Spontaneous LDH and Maximum LDH samples in relation to the number of EPC cells

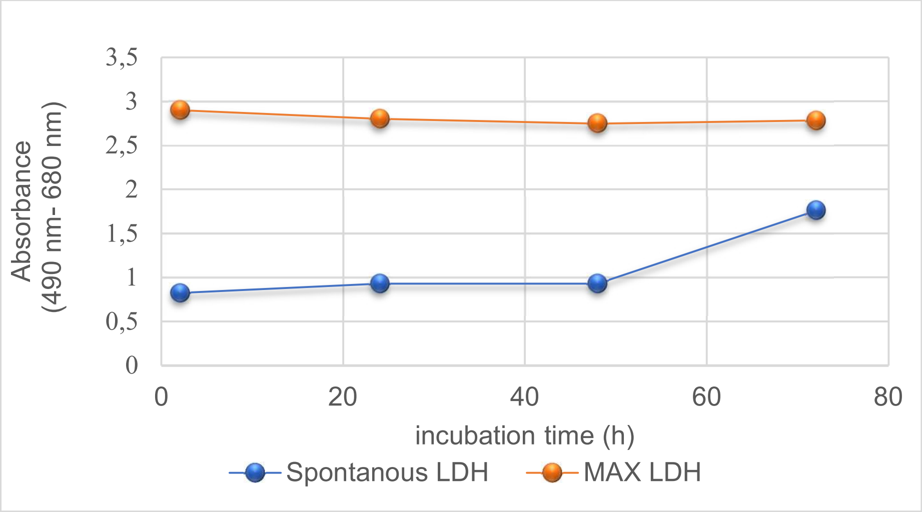

Supplementary Fig. S2. The absorbance values for Spontaneous LDH and Maximum LDH samples relation to the incubation time
